# Improving vasculoprotective effects of MSCs in coronary microvessels – benefits of 3D culture, sub-populations and heparin

**DOI:** 10.3389/fimmu.2023.1257497

**Published:** 2023-10-24

**Authors:** Kobkaew Bumroongthai, Dean P. J. Kavanagh, Paul Genever, Neena Kalia

**Affiliations:** ^1^ Microcirculation Research Group, Institute of Cardiovascular Sciences, College of Medical and Dental Sciences, University of Birmingham, Birmingham, United Kingdom; ^2^ Department of Biology, University of York, York, United Kingdom

**Keywords:** myocardial infarction, myocardial ischaemia-reperfusion injury, coronary microcirculation, mesenchymal stem/stromal cells, intravital microscopy, neutrophils, platelets

## Abstract

**Introduction:**

Opening occluded coronary arteries in patients with myocardial infarction (MI) damages the delicate coronary microvessels through a process called myocardial ischaemia-reperfusion injury. Although mesenchymal stromal cells (MSCs) have the potential to limit this injury, clinical success remains limited. This may be due to (i) poor MSC homing to the heart (ii) infused MSCs, even if derived from the same site, being a heterogeneous population with varying therapeutic efficacy and (iii) conventional 2D culture of MSCs decreasing their homing and beneficial properties. This study investigated whether 3D culture of two distinctly different bone marrow (BM)-derived MSC sub-populations could improve their homing and coronary vasculoprotective efficacy.

**Methods:**

Intravital imaging of the anaesthetised mouse beating heart was used to investigate the trafficking and microvascular protective effects of two clonally-derived BM-derived MSC lines, namely CD317^neg^ MSCs-Y201 and CD317^pos^ MSCs-Y202, cultured using conventional monolayer and 3D hanging drop methods.

**Results:**

3D culture consistently improved the adhesive behaviour of MSCs-Y201 to various substrates *in vitro*. However, it was their differential ability to reduce neutrophil events within the coronary capillaries and improve ventricular perfusion *in vivo* that was most remarkable. Moreover, dual therapy combined with heparin further improved the vasculoprotection afforded by 3D cultured MSCs-Y201 by also modifying platelet as well as neutrophil recruitment, which subsequently led to the greatest salvage of viable myocardium. Therapeutic benefit could mechanistically be explained by reductions in coronary endothelial oxidative stress and intercellular adhesion molecule-1 (ICAM-1)/vascular cell adhesion molecule-1 (VCAM-1) expression. However, since this was noted by both 2D and 3D cultured MSCs-Y201, therapeutic benefit is likely explained by the fact that 3D cultured MSCs-Y201 were the most potent sub-population at reducing serum levels of several pro-inflammatory cytokines.

**Conclusion:**

This novel study highlights the importance of not only 3D culture, but also of a specific CD317^neg^ MSC sub-population, as being critical to realising their full coronary vasculoprotective potential in the injured heart. Since the smallest coronary blood vessels are increasingly recognised as a primary target of reperfusion injury, therapeutic interventions must be able to protect these delicate structures from inflammatory cells and maintain perfusion in the heart. We propose that relatively feasible technical modifications in a specific BM-derived MSC sub-population could achieve this.

## Graphical Abstract

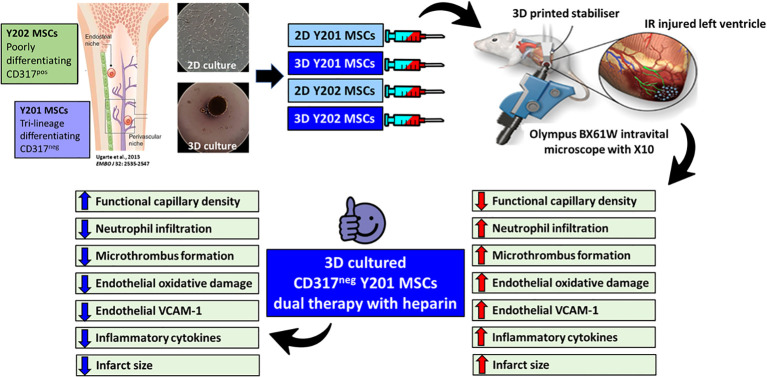

## Introduction

Treatment of myocardial infarction (MI) focuses on rapidly re-establishing reperfusion following blockage in one or more coronary arteries. Despite successful thrombolytic and primary percutaneous coronary interventions (PCI) a significant number of patients still subsequently proceed to develop muscle damage and heart failure (1–3). This is partly due to reperfusion paradoxically leading to additional tissue damage, a condition termed ischaemia-reperfusion (IR) injury (4, 5). Since blood flow is normally restored in the occluded artery, tissue damage most likely occurs as a consequence of inadequate coronary microcirculatory perfusion (6). Indeed, sub-optimal myocardial perfusion can be observed in as high as 50% of patients, leading to worse outcomes than in patients with full perfusion recovery (7, 8). Increased clinical recognition of the importance of the coronary microcirculation has meant identifying strategies to improve detrimental perturbations within it have gained recent attention (9, 10). However, clinical imaging tools cannot spatially resolve blood vessels <200μm in diameter and so little is known about the full range of coronary microcirculatory responses to IR injury and whether potential strategies are vasculoprotective (11, 12). Moreover, *in vivo* experimental studies of the beating heart using intravital imaging have also been hampered due to obvious difficulties related to significant inherent contractile motion, movement of the heart brought about by nearby lungs and its location in an anatomically challenging position for microscopy (13).

Recently, we described an intravital microscopy technique that allowed imaging of the coronary microcirculation of the mouse beating heart with cellular resolution *in vivo* (14, 15). Using this technique, we showed that despite deceptive hyperaemic responses, increased microcirculatory flow heterogeneity and reduced functional capillary density (FCD) was observed post-reperfusion. This was primarily linked to marked neutrophil infiltration and occlusive platelet aggregate formation within coronary capillaries which consequently impacted infarct size. Therefore, therapeutic strategies that can ameliorate these perturbations and restore microcirculatory perfusion in the immediate aftermath of reperfusion are essential if salvaging viable myocardium is to be maximized.

Mesenchymal stromal cells (MSCs) have been at the forefront as candidates to treat cardiovascular diseases (CVDs). (16) This has been due to the ease with which they can be isolated and expanded in culture but mainly because of their potent anti-inflammatory and immunomodulatory capabilities. However, despite evidence that MSCs are beneficial in experimental models of myocardial IR injury, clinical trials have either failed to replicate these findings or have shown minor or temporary benefit. Therapeutic success is likely dependent on the ability of systemically injected cells to successfully migrate or home to the ischaemic heart and become retained by the local coronary microcirculation (17, 18). However, myocardial MSC homing is inefficient which may prevent their full therapeutic potential from being realised, necessitating the need to develop strategies to improve their recruitment (17, 18). Limited therapeutic success may also be attributable to increasing evidence demonstrating a significant heterogeneity, both morphologically and functionally in terms of differentiation and migratory capacity, amongst MSCs even if they are derived from the same tissue site (19). Recent studies have also suggested that 3D spheroid culture of MSCs utilising techniques such as non-adherent dishes, cell sheets, micro-fluidics and the hanging drop method, may be necessary to enhance their beneficial properties for CVDs (20, 21). Indeed, 3D cultured MSCs exhibit greater anti-inflammatory potential, stemness, angiogenic properties and survival rates in ischaemic tissue following their systemic administration (20, 22, 23).

Whether there is a difference in the myocardial homing ability and vasculoprotective efficacy of 2D or 3D cultured intra-tissue MSC sub-populations has not previously been investigated in any tissue let alone the heart, but may prove critical to improving outcomes for MI patients in the acute period post-PCI. To investigate this, the current study used two MSC sub-populations, both derived from the bone marrow (BM), with distinct phenotype and functionality and characterised by CD317 expression (24, 25). MSCs-Y201 are CD317^neg^ and exhibit potent tri-lineage differentiation capabilities. They also have a higher expression set of genes related to interactions with BM vasculature and in playing a role in vascular processes such as blood vessel remodelling, morphogenesis, development and patterning. In contrast, MSCs-Y202 are CD317^pos^, poorly or non-differentiating and rarely associate with vascular cells or perivascular stroma (24, 25). Both sub-populations were cultured using conventional 2D and spheroid forming 3D methods and their ability to home to, become retained within, and subsequently vasculoprotect the coronary microcirculation was investigated intravitally in the IR injured mouse beating heart *in vivo*.

## Methods

### 2D and 3D culture of BM-derived MSC sub-populations

Primary MSCs were isolated from a single anonymous patient undergoing routine hip or knee replacement after obtaining written informed consent (REC 07/Q1105/9; University of York). Whilst iliac crest aspirated MSCs may be preferred due to their high yield of cells, distal/proximal femur and tibia from replacement surgery provides a common, non-invasive, relatively easy-to-obtain and high-yield source of MSCs. MSCs-Y201 and MSCs-Y202 were subsequently expanded by cell cloning and telomerase-based immortalization as previously described (24). Both sub-populations were cultured either using conventional 2D culture conditions or a 3D sphere forming hanging drop method (26). For the latter, 35μl droplets, which consisted of approximately 25,000 cells, were pipetted onto the inverted lid of a 150mm petri dish. This was then inverted over a phosphate buffered saline (PBS) filled petri dish and incubated at 37°C with 5% CO_2_ for 1 or 2 days. To measure cell size, 2D and 3D cultured MSCs were dissociated using Accutase^®^ (Stem Cell Technologies, UK) to create a single cell suspension and fluorescently labelled with 5uM carboxyfluorescein succinimidyl ester (CFSE; Life Technologies, UK). Cells were imaged microscopically (EVOS; Thermo Fisher Scientific) at 1 and 2 days cultivation and their diameter determined using ImageJ software (NIH; USA).

### Flow cytometric and confocal microscopy analysis of MSC surface adhesion molecules

After 2 days culture, dissociated 2D and 3D cultured MSCs were incubated with mouse anti-human fluorescein isothiocyanate (FITC) conjugated anti-CXCR4, rat anti-human FITC conjugated anti-CD44, mouse anti-human FITC conjugated anti-ICAM-1 and mouse anti-human FITC conjugated anti-VCAM-1 (all at 1:100; all from eBiosciences). After washing and resuspending in FACS buffer, 10,000 events were analysed flow cytometrically (FACSCalibur; Beckman Coulter) with data visualised using Flowing software 2.5.1 (University of Turku, Finland). Adhesion molecule expression was presented as a percentage of the total cell count. Confocal analysis of the CD44 and ICAM-1 was also performed on formalin fixed MSCs as previously described using rat anti-human FITC conjugated anti-CD44 and mouse anti-human FITC conjugated anti-ICAM-1 (all at 2:25 dilution; eBiosciences) (27). A laser scanning confocal microscope (Zeiss LSM Zen 780, USA) with a 40x magnification water immersion lens was used to scan slides and obtain images for qualitative analysis.

### Static adhesion assays on frozen heart tissue, endothelial cells and ICAM-1/VCAM-1

The adhesive capacity of 2D and 3D cultured MSCs to various substrates was tested *in vitro* including on snap frozen sections of sham or IR injured mouse heart tissue, PBS or tumour necrosis factor-α (TNFα) stimulated murine vena cava endothelial cells (VCECs) and immobilised ICAM-1 or VCAM-1 as previously described (27). Briefly, 1 x 10^5^ CFSE-labelled MSCs were added to 8µm frozen heart sections for 1 hour at 37°C, PBS washed to remove non-adherent cells and then slides were acetone fixed. To test adhesion specifically to ECs, 25,000 VCECs were grown on gelatin coated 24-well plates until confluent and then stimulated for 4 hours with TNFα (100ng/ml; Life Technologies, UK) or the vehicle control PBS. 5 x 10^4^ CFSE-labelled MSCs were then incubated with VCECs for 20 minutes and PBS washed to remove non-adherent cells. For immobilised protein assays, 96-well plates were coated for 1 hour at 37°C with recombinant murine ICAM-1 or VCAM-1 (10mg/ml; R&D Systems, UK). Plates were washed with PBS and blocked with 1% bovine serum albumin (Sigma, UK). 5 x 10^4^ CFSE-labelled MSCs were then added to each well, incubated for 20 minutes at 37°C, PBS washed to remove non-adherent cells and fixed with 2% glutaraldehyde. Adherent MSCs on all substrates were imaged using an EVOS microscope and manually counted in 10 or 20 randomly selected fields of view.

### Myocardial ischaemia-reperfusion injury

Experiments were conducted on 8–12-week-old male C57Bl/6 mice in accordance with the Animals (Scientific Procedures) Act of 1986 (Project license P552D4447 and P95F9B96). Anaesthesia was induced using ketamine hydrochloride (100mg/kg) and medetomidine hydrochloride (100mg/kg) (Vetoquinol, UK) and maintained as required via further intraperitoneal administration. Mice were intubated and ventilated with medical oxygen using a MiniVent rodent ventilator (stroke volume: 220 μL, respiratory rate: 130 breaths/min; Biochrom Ltd., Harvard Apparatus). The carotid artery was cannulated to facilitate administration of MSCs, fluorescent dyes, heparin and saline. IR injury was induced by ligating the left anterior descending (LAD) artery for 45 minutes and reperfusion was allowed to proceed for 2 hours (tissue analysis and intravital observations) or 4 hours (infarct measurement) by removal of the suture. Sham surgery involved the same procedure but without ligation. At the end of experiments, mice were euthanised by cervical dislocation.

### Intravital imaging of the beating heart coronary microcirculation *in vivo*

Real-time intravital imaging of the beating heart was performed as previously described (14, 15). Briefly, an in-house designed 3D-printed stabilizer was attached to the surface of the left ventricle downstream of the ligation site. To track MSC homing and recruitment, a bolus dose of 5 x 10^5^ CFSE-labelled MSCs were systemically infused at 30 minutes post-reperfusion. Two-minute-long recordings were taken of a randomly selected area within the stabiliser centre immediately after MSC infusion and then every 15 minutes for 2 hours. At the end of imaging, heart and lungs were removed from culled mice, digested using 0.1% collagenase for 1 hour at 37˚C to obtain a single cell suspension and analysed flow cytometrically (FACSCalibur; Beckman Coulter) for CFSE-labelled MSCs.

To investigate the impact of cellular therapy on neutrophil and platelet presence in the coronary microcirculation, neutrophils were labelled with a PE-conjugated anti-mouse Ly-6G antibody (0.2mg/ml; BioLegend) and platelets with an APC-conjugated anti-mouse CD41 antibody (0.2mg/ml; BioLegend). Two-minute-long recordings of a randomly selected area were captured at 30 minutes post-reperfusion and then every 15 minutes for 2 hours. FITC-BSA (Sigma, UK) was injected at the end of the 2-hour reperfusion period to investigate overall microvascular perfusion. FCD was quantitated using Image J and presented as the perfused vascular area in pixels. In some mice, 400 IU unfractionated heparin sodium salt (Sigma, UK) was infused on its own or as a dual therapy with MSCs at 30 minutes post-reperfusion.

Intravital imaging was performed using a microscope (BX61WI, Olympus) equipped with a Nipkow spinning disk confocal head (Yokogawa CSU) and an Evolve EMCCD camera (Photometrics). Data was captured, stored and analysed using Slidebook 6 software (Intelligent Imaging Innovations, USA). Free-flowing MSCs were manually counted as those cells that passed through the coronary microcirculation during the 2-minute recording time without making firm adhesive interactions. Adherent MSCs were quantitated manually as those cells that remained stationary within the microvessel for ≥ 30 seconds during the 2-minute recording period. To analyse adherent neutrophil and platelet presence, captured videos were subjected to post-acquisition image repair using an in-house designed software (*Tify*) in which out-of-focus frames were firstly removed (28). Neutrophils were then counted manually as for MSCs and platelet aggregates/microthrombi were quantitated by placing a mask around APC-CD41^+^ areas. Integrated fluorescence density, which considered both the size and fluorescence intensity of the platelets, was then calculated using ImageJ software.

### Multiphoton imaging of heart sections

Intravital study of the beating heart is restricted to continuously imaging one pre-selected region with a depth of approximately 50-60μm. To determine whether these captured events were mirrored throughout the thickness of the ventricular wall, multiphoton microscopy was performed on hearts harvested at the end of intravital experimentation. 50 IU heparin was infused intra-arterially immediately before culling to prevent post-mortem platelet coagulation. The left ventricle was vibratome (Campden Instruments Limited, UK) sectioned into four 250µm sections and imaged from the outermost to the innermost chamber side using a multiphoton microscope (FVMPE-RS Olympus). Z-stacks were collected from the four sections with images rendered to create 3D stacks that were processed and displayed using ImageJ software. The presence of neutrophils and platelets was analysed as the sum of the mean fluorescence intensity (MFI) in each image.

### Myocardial infarct size analysis

At 4 hours post-reperfusion, the LAD artery was re-ligated and Evans blue (Sigma, UK) was infused via the carotid artery to identify the area at risk (AAR). The mouse was then sacrificed and the harvested heart was cut into slices and incubated with 2,3,5-triphenyltetrazolium chloride (TTC, Sigma, UK). Formalin-fixed hearts were then imaged using a stereomicroscope and analysis was performed using ImageJ software to quantitate the infarct size (TTC^neg^ white regions) as a percentage of the AAR (TTC^pos^ red regions/Evans blue^neg^ regions).

### Flow cytometric analysis of endothelial oxidative injury and surface adhesion molecules

The ability of 2D and 3D cultured MSCs to modify reactive oxygen species (ROS) generation and protect VCECs from oxidative damage after ROS exposure was determined by immunostaining using dihydroethidium (DHE) and an anti-8-OHdG antibody respectively. DHE is oxidised to 2-hydroethidium specifically in the presence of the ROS superoxide, which is further converted to ethidium that intercalates with nucleic acids staining them a bright fluorescent red. 8-OHdG is a marker of oxidative DNA damage produced specifically by ROS (14). Briefly, VCECs were challenged for 24 hours with 10μM hydrogen peroxide (Sigma, UK) in the presence or absence of 5 x 10^4^ MSCs and subsequently fixed and permeabilised with ethanol. To block non-specific binding of IgG to Fc receptors, 300µl of the blocking agent anti-CD16/CD32 (1:500; BioLegend) was added at room temperature for 30 minutes. After washing, cells were incubated with DHE (500μl of 10μM; Sigma) or a goat anti-mouse 8-OHdG antibody (1:500; Abcam) followed by the secondary Alexa Fluor 488 donkey anti-goat antibody (1:250; Abcam). Images were captured using an EVOS microscope and the MFI determined.

The ability of MSCs to decrease oxidative damage of coronary ECs and cardiomyocytes derived from mice undergoing IR injury was also determined. Harvested hearts were 0.1% collagenase digested, centrifuged in Dulbecco’s modified eagle medium at 2000 rpm for 10 minutes and the pellet permeabilised and fixed with ethanol. Cells were then incubated with Alexa Fluor 488-conjugated IgG (1:500; Abcam), PE-conjugated anti-CD31 (1:50; BioLegend), PE-conjugated IgG2a control (1:50; BioLegend), PE-conjugated REA control antibody (1:10; Miltenyi Biotech, UK), PE-conjugated anti-cTnT (1:10; Miltenyi Biotech) and Alexa Fluor 488-conjugated anti-8-OHdG (1:500; Abcam). Changes in ICAM-1 and VCAM-1 expression on ECs was also determined using PE-conjugated anti-CD31 (1:50; BioLegend UK), PE-conjugated IgG2a control (1:50; BioLegend), FITC-conjugated anti-ICAM-1 (1:50, BioLegend), FITC-conjugated IgG2a control (1:50; BioLegend) PECy7-conjugated anti-VCAM-1 (1:50; BioLegend) and PECy7-conjugated IgG2b control (1:5; BioLegend). 25,000 events were analysed flow cytometrically with data visualised using Summit 4.3 software (Beckman Coulter).

### Luminex^®^ multiplex immunoassay to assess circulating cytokine levels

Luminex^®^ multiplex immunoassay was used to investigate the serum levels of 23 different pro- and anti-inflammatory cytokines. 500μl of anti-coagulated blood was collected from sacrificed mice at the end of 2 hours reperfusion and centrifuged at 2000 rpm to obtain serum. Samples were loaded in triplicate onto a BIO RAD^®^ Bio-Plex Pro mouse cytokine 23-plex assay plate and analysed using Bio-Plex MAGPIX^®^ plate reader (Bio-Rad Laboratories; USA).

### Statistical analysis

Statistical analysis was performed using GraphPad 7.0 software. All data was firstly tested for normality using a Shapiro-Wilk test and a subsequent parametric test was only performed if the data passed this test for normality. Direct comparisons between two groups were performed using a Student’s unpaired t-test. Multiple comparisons between three or more groups were performed by 1- or 2-way analysis of variance (ANOVA), followed by a Tukey or Sidak’s *post-hoc* test. For experiments which followed a time course, the area under the curve (AUC) was also calculated and used for subsequent analysis as a summation of the entire period. All data are presented as mean ± SEM with statistical significance defined when p<0.05.

## Results

### 3D culture reduced the cell diameter of both MSC sub-populations

**Figure 1:**
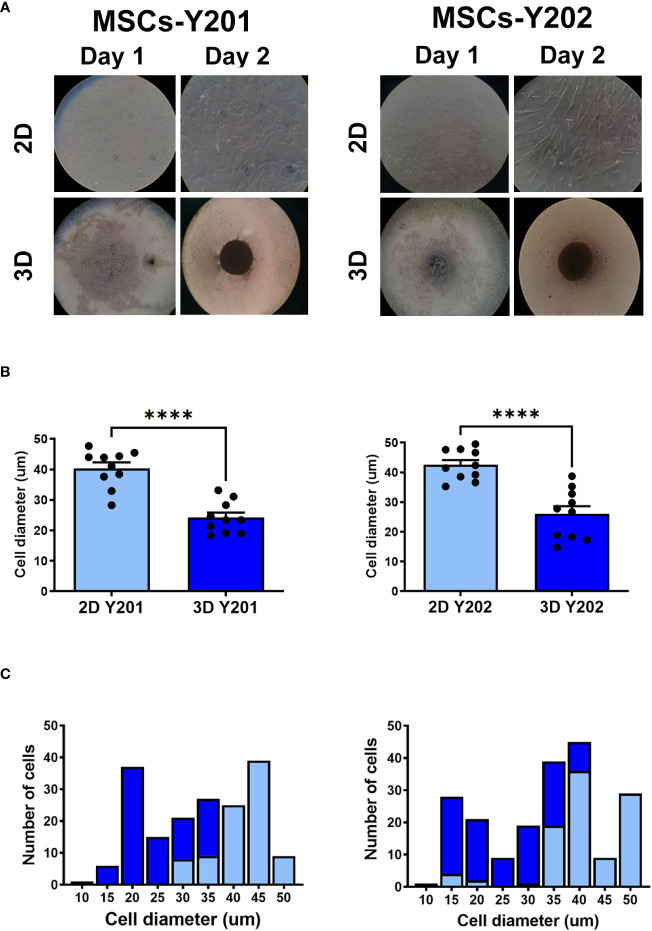
Effects of 2D/3D culture on MSC morphology and size distribution. **(A)** Appearance of 2D/3D cultured MSCs after 1 and 2 days of culture. **(B)** Cell diameter and **(C)** cell size distribution of 2D and 3D cultured MSCs after their removal from culture flasks. Results presented as mean ± SEM. ****p<0.0001 with a Student’s t-test; N=10 with 50 cells in each group.

2D cultured MSCs-Y201 and MSCs-Y202 were adherent, elongated and fibroblastic in appearance by day 2 of culture. However, 3D cultured cells had clumped together and formed a compact spheroid of more rounded cells (**Figure 1A**). Both 3D cultured sub-populations were significantly (p<0.0001) smaller than those grown in 2D culture when their cell diameter was measured following their removal from culture flasks. The average cell diameter of 2D and 3D cultured MSCs-Y201 was 40.4 ± 1.93µm and 24.2 ± 1.63µm respectively. For 2D and 3D cultured MSCs-Y202 it was 42.5 ± 1.62µm and 26.0 ± 2.63µm respectively (**Figures 1B, C**).

### 3D culture increased CXCR4, CD44 and/or ICAM-1 expression on MSC sub-populations

**Figure 2:**
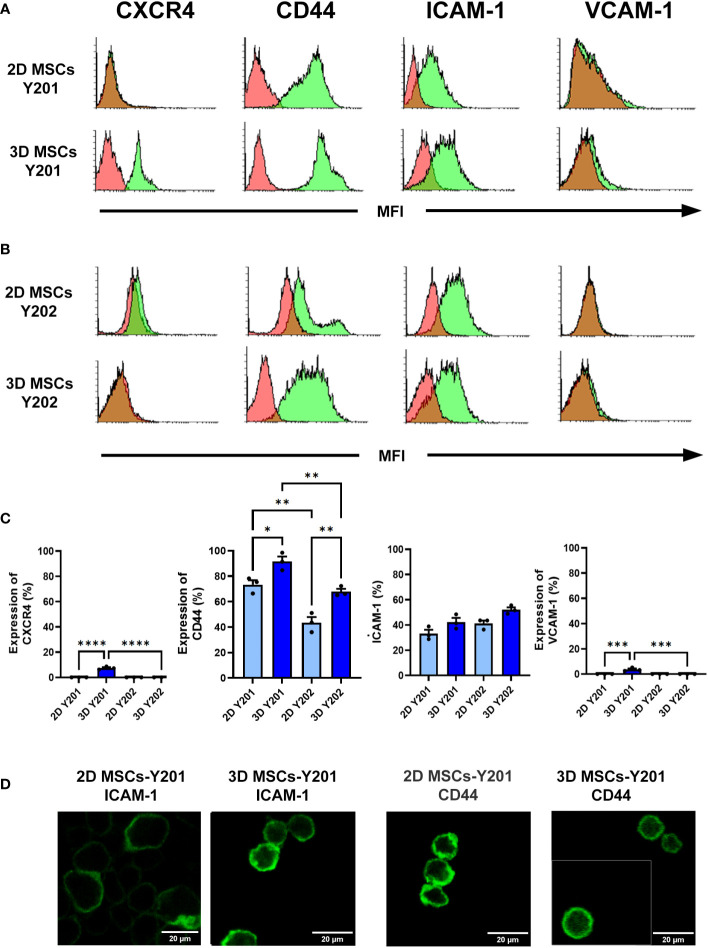
Effects of 2D/3D culture on MSC expression of surface molecules. Flow cytometry was performed on dissociated MSCs at after 2 days of culture. Expression of CXCR4, CD44, ICAM-1 and VCAM-1 on **(A, C)** 2D/3D cultured MSCs-Y201 and **(B, C)** MSCs-Y202. Green filled histograms = 2D/3D cultured MSCs; red filled histograms = isotype controls. Analysis conducted on 10,000 events. Samples were analyzed using FACSCalibur flow cytometry with analysis conducted on 10,000 events. **(D)** Confocal microscopy images taken after 2 days of culture. Representative images are shown for surface expression of ICAM-1 and CD44 on 2D and 3D MSCs-Y201 and 2D and 3D MSCs-Y202 respectively. Results presented as mean ± SEM. *p<0.05, **p<0.01, ***p<0.001, ****p<0.0001 with ANOVA and Tukey’s multiple comparison test. Scale bar = 20 μm.

2D cultured MSCs-Y201 did not express CXCR4 or VCAM-1 but did express CD44 and ICAM-1. Although expression of all four increased with 3D culture, this was only statistically significant for CXCR4 (p<0.0001), CD44 (p<0.05) and VCAM-1 (p<0.001) (**Figures 2A, C**). Similarly, 2D cultured MSCs-Y202 did not express CXCR4 or VCAM-1 but did express CD44 and ICAM-1. 3D culture only increased expression of CD44 (p<0.01) and ICAM-1, although the latter did not attain statistical significance (**Figures 2B, C**). Interestingly, 2D (p<0.01) and 3D (p<0.01) cultured MSCs-Y201 had significantly higher levels of CD44 when compared to MSCs-Y202 (**Figures 2A–C**). Since flow cytometry only demonstrated basal CD44 and ICAM-1 expression on both MSC sub-types with 2D culture, we further assessed the cell surface distribution of these adhesion molecules confocally. CD44 was uniformly expressed on the surface of MSCs-Y201 regardless of culture technique. However, although ICAM-1 on 2D cultured MSCs-Y201 tended to be concentrated on one side of the cell surface, it was more evenly distributed over the entire cell surface after 3D culture. In contrast, ICAM-1 was uniformly expressed on the surface of MSCs-Y202 regardless of culture technique. However, CD44 was concentrated on one side of the cell surface on 2D cultured MSCs-Y202 but was more evenly distributed over the entire cell surface after 3D culture (**Figure 2D**).

### 3D culture consistently improved adhesion of MSCs-Y201 in static adhesion assays *in vitro*

**Figure 3:**
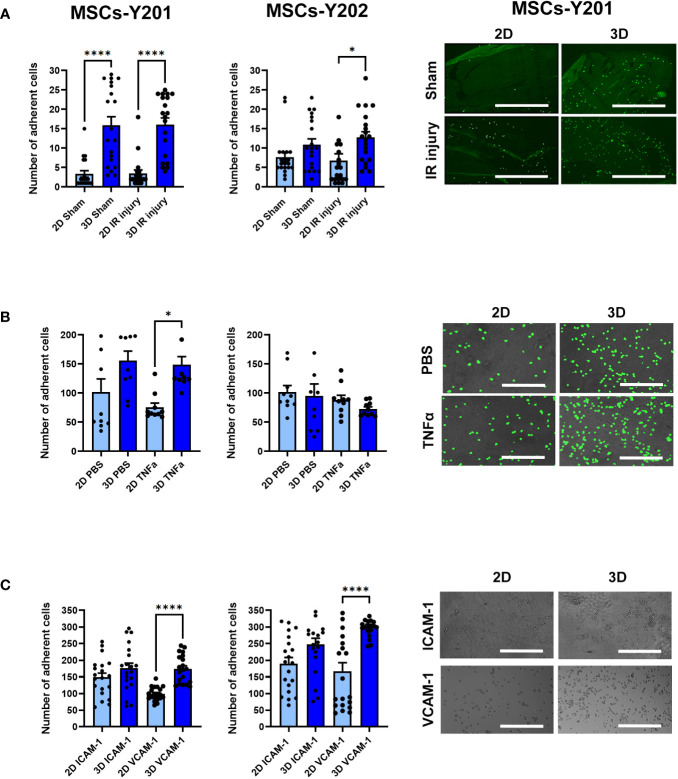
Effects of 2D/3D culture on MSC adhesion in static adhesion assays. **(A)** MSC adhesion on frozen sections of sham/IR injured heart tissue with representative images showing MSCs-Y201 adhesion. N=5 mice with 20 fields of view counted. **(B)** MSC adhesion on PBS/TNFα stimulated VCECs with representative images show MSCs-Y201 adhesion. 10 fields of view/group with each group conducted in triplicate. **(C)** MSC adhesion on ICAM-1/VCAM-1 with representative images showing MSCs-Y201 adhesion. 20 fields of view/group with each group conducted in triplicate. Results presented as mean ± SEM. *p<0.05, ****p<0.0001 with ANOVA and Tukey’s multiple comparison test. Scale bar = 1000μm.

Adhesion of 3D cultured MSCs-Y201 on frozen sections of sham (p<0.0001) and IR injured (p<0.0001) mouse heart tissue was significantly improved when compared to 2D cultured cells. Adhesion of 3D cultured MSCs-Y202 was only significantly (p<0.05) increased on IR injured heart tissue (**Figure 3A**). Adhesion was not directed towards any particular cell type and occurred on vessels and cardiomyocytes. Although adhesion of 3D cultured MSCs-Y201 increased on PBS and TNFα stimulated VCECs, this was only significant (p<0.01) for the latter. No differences were observed for 3D cultured MSCs-Y202 on VCECs (**Figure 3B**). Adhesion of 3D cultured MSCs-Y201 (p<0.0001) and MSCs-Y202 (p<0.0001) significantly increased on immobilised VCAM-1, with no significant increases for either on ICAM-1 (**Figure 3C**).

### 3D culture improved adhesion of MSCs-Y201 in the IR injured beating heart *in vivo*

**Figure 4:**
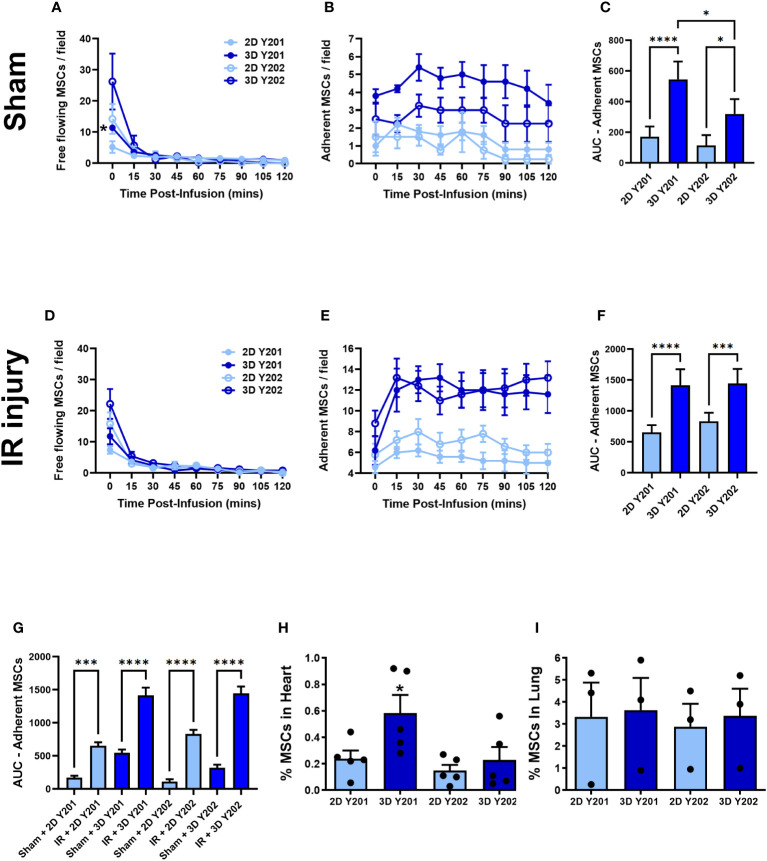
Effects of 2D/3D culture on MSC trafficking and adhesion within sham and IR injured beating hearts *in vivo.* **(A)** Free flowing MSCs **(B)** adherent MSCs and **(C)** AUC analysis of the continuous intravital data for adherent MSCs in sham hearts. **(D)** Free flowing MSCs **(E)** adherent MSCs and **(F)** AUC analysis of the continuous intravital data for adherent MSCs in injured hearts. **(G)** AUC analysis of the continuous intravital data for adherent MSCs in both sham and injured hearts. Flow cytometric analysis of digested **(H)** hearts and **(I)** lungs isolated from mice undergoing myocardial IR injury to quantitate fluorescent MSC presence. Analysis was conducted on 100,000 events with the percentage of MSCs present calculated by the number identified divided by the total number of infused MSCs. Results presented as mean ± SEM. *p<0.05, ***p<0.001; ****p<0.0001 with ANOVA and Tukey’s multiple comparison test. N=5 for all groups; N=3 for *ex vivo* lung data.

Intravital imaging demonstrated that 3D culture increased numbers of MSCs-Y201 and MSCs-Y202 freely flowing through sham hearts when compared to 2D cultured cells. However, this only happened immediately after their infusion with a significant (p<0.05) difference only noted for 3D cultured MSCs-Y201. Thereafter, limited MSCs were intravitally observed circulating through the coronary microcirculation (**Figure 4A**). A similar pattern of freely flowing MSCs was observed in injured hearts (**Figure 4D**). 3D culture significantly increased adhesion of MSCs-Y201 (AUC; p<0.0001) and MSCs-Y202 (AUC; p<0.05) at all time points within sham hearts when compared to 2D cultured cells. Moreover, 3D cultured MSCs-Y201 were retained within sham hearts in significantly (AUC; p<0.05) greater numbers than 3D cultured MSCs-Y202 (**Figures 4B, C**). A similar pattern for MSC adhesion was noted in injured hearts (**Figures 4E, F**). Also, injury significantly (AUC; p<0.001-0.0001) increased MSC adhesion for all sub-populations regardless of culture technique when compared to sham hearts (**Figure 4G**). Flow cytometric analysis of digested heart tissue confirmed a significant (p<0.05) increase in myocardial retention of 3D cultured MSCs-Y201 *ex vivo* in injured hearts. However, most infused MSCs were retained in the lungs, which was not affected by sub-population type or culture technique (**Figures 4H, I**).

### 3D cultured MSCs-Y201 were most vasculoprotective in the IR injured beating heart *in vivo*

**Figure 5:**
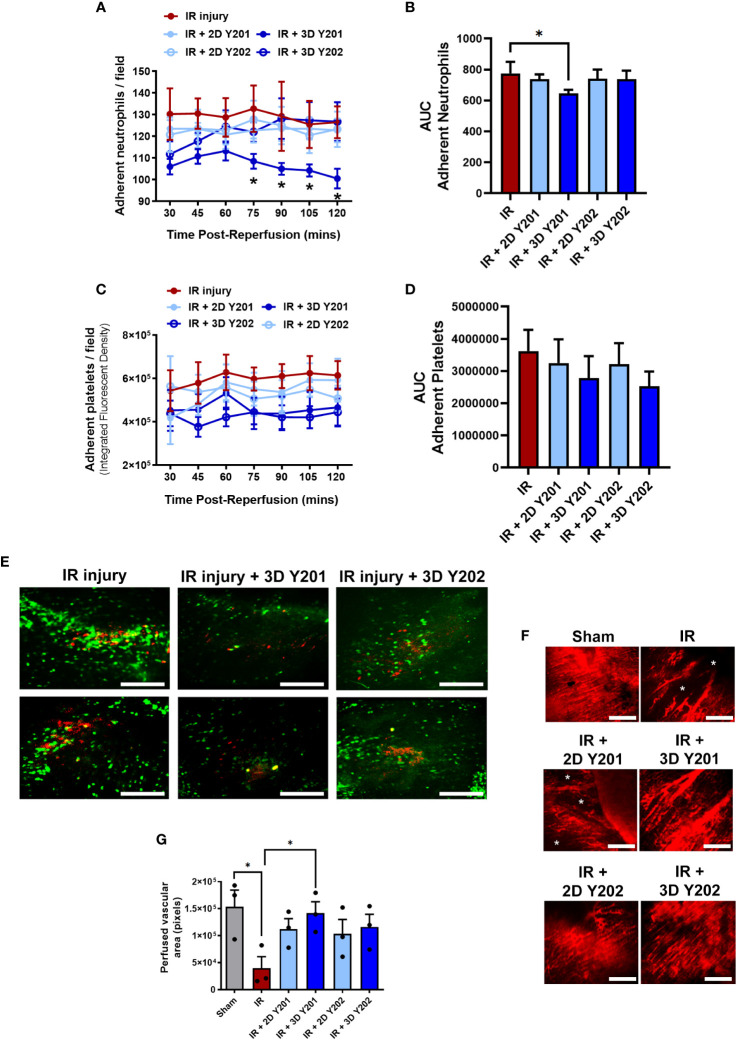
Effects of 2D/3D culture of MSCs on neutrophil/platelet adhesion and functional capillary density in IR injured hearts *in vivo.* **(A)** Adherent neutrophils and **(B)** AUC analysis of the continuous intravital data for neutrophil adhesion. **(C)** Adherent platelets and **(D)** AUC analysis of the continuous intravital data for platelet adhesion. **(E)** Representative intravital images demonstrating neutrophil (green; PE-conjugated anti-Gr-1/Ly6G antibody) and platelet (red; APC-conjugated anti-CD41 antibody) recruitment within the IR injured beating heart at 120 minute post-reperfusion. Platelets formed either pure platelet-platelet (red) or mixed platelet-neutrophil aggregates (yellow). The presence of these mixed aggregates was limited in comparison to infiltration of neutrophils. Whether this co-localisation presents *bona fide* complexes of these two cells, or is due to neutrophils gathering behind occlusive microthrombi and/or *vice versa*, is impossible to ascertain. **(F)** Representative intravital images demonstrating a dense network of FITC-BSA perfused coronary capillaries in sham mice and multiple areas in which FITC-BSA did not perfuse, resulting in patchy areas devoid of vasculature (*) in untreated and some treated injured hearts. **(G)** Analysis of functional capillary density. Results presented as mean ± SEM. *p<0.05 with 2-way ANOVA and Sidak’s multiple comparison test. N=5 per group with N=3 for FITC-BSA experiments. Scale bar = 200μm.

Neutrophil and platelet microthrombi were present within coronary capillaries by the time of the first intravital imaging at 30 minutes post-reperfusion. Only 3D cultured MSCs-Y201 significantly (AUC; p<0.05) reduced this neutrophil adhesion. Detailed analysis of the continuous data showed that this was only significant (p<0.05) from 75 minutes onwards when compared to untreated mice. Indeed, adherent neutrophil numbers noted at the end of imaging decreased to ∼100 cells/FOV in 3D cultured MSCs-Y201 treated mice compared to ∼130 cells/FOV in untreated mice (**Figures 5A, B, E**). Although 3D cultured MSCs also reduced platelet presence, this did not attain statistical significance in these intravital studies (**Figures 5C–E**). Interestingly, whilst neutrophils (green) presented primarily as individual cells, and platelets as aggregates or microthrombi (red), platelet-neutrophil complexes were also visible (yellow). However, the presence of these mixed aggregates was very much limited in comparison to pure neutrophil or pure platelet recruitment (**Figure 5E**).

An extensive network of FITC-BSA perfused capillaries was observed in sham mice. Focusing up and down on the field of view showed no areas devoid of perfused capillaries. However, in IR injured hearts, multiple areas were detected in which FITC-BSA did not perfuse. This resulted in patchy areas without perfusion and was indicative of significantly (p<0.05) reduced FCD. In some areas, up to half the imaged area appeared non-perfused. Interestingly, medium sized vessels were still readily visible and well perfused even in injured hearts. Treatment with 3D cultured MSCs-Y201 was most effective at significantly (p<0.05) improving myocardial FCD when compared to non-treated hearts (**Figures 5F, G**).

### Dual therapy induced an earlier onset of anti-inflammatory effects and reduced platelet presence in the IR injured beating heart *in vivo*

**Figure 6:**
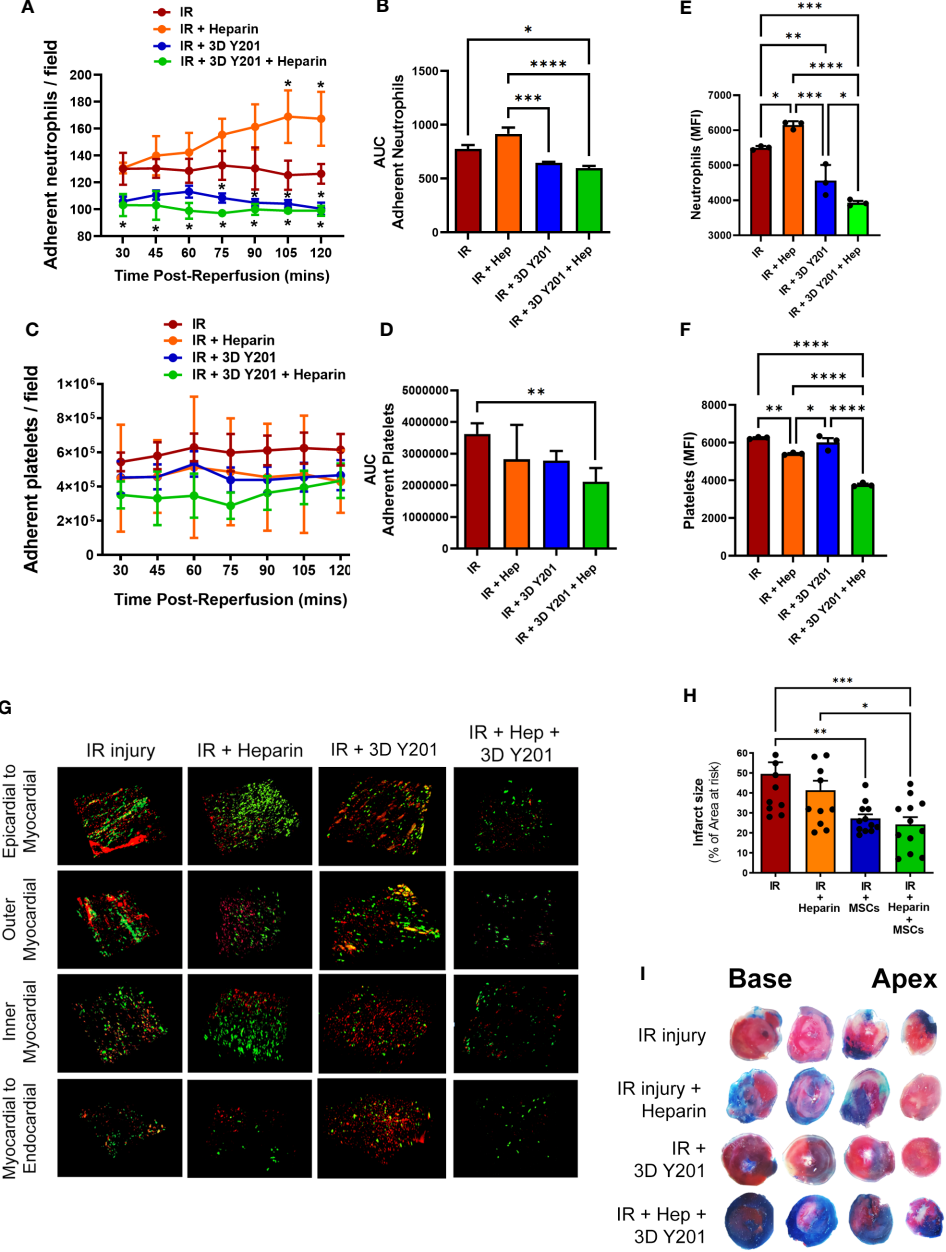
Effects of dual therapy with heparin and MSCs-Y201 on neutrophil/platelet adhesion in IR injured hearts *in vivo.* **(A)** Adherent neutrophils and **(B)** AUC analysis of the continuous intravital data for neutrophil adhesion. **(C)** Adherent platelets and **(D)** AUC analysis of the continuous intravital data for platelet adhesion. Multiphoton microscopy analysis of **(E)** neutrophil and **(F)** platelet presence in all 4 layers of the heart. **(G)** Representative multiphoton images of neutrophils (green; PE-conjugated anti-Gr-1/Ly6G antibody) and platelets (red; APC-conjugated anti-CD41 antibody) in all 4 layers of the injured heart ventricle. **(H)** Infarct size as a percentage of the area at risk. **(I)** Representative images of dual Evans blue and TTC staining showing non-ischaemic area (blue), area of the viable tissue at risk (red) and infarct area (white) after IR injury. Results presented as mean ± SEM. *p<0.05, **p<0.01, ***p<0.001, ****p<0.0001 with 2-way ANOVA and Sidak’s multiple comparison test. N=5 per group.

Since no significant anti-platelet effect was observed, we investigated whether co-transplantation of 3D cultured MSCs-Y201, the most effective anti-inflammatory sub-population, with the anti-coagulant heparin could improve their ability to modify platelet as well as neutrophil presence. Interestingly, heparin alone increased adherent neutrophil number over time with significant (p<0.05) increases noted at 105 and 120 minutes post-reperfusion when compared with untreated injured hearts. Indeed, adherent neutrophils at the end of imaging increased to ∼170 cells/FOV in heparin alone treated mice compared to ∼130 cells/FOV in untreated mice. However, co-administration of heparin with 3D cultured MSCs-Y201 significantly (AUC; p<0.05) decreased neutrophil recruitment when compared to untreated mice. This significant (p<0.05) anti-neutrophil effect was observed at all time points post-reperfusion, unlike with 3D cultured MSCs-Y201 alone when it was only noted after 75 minutes of reperfusion (**Figures 6A, B**). In contrast to cellular therapy alone, dual therapy with heparin also significantly (AUC; p<0.01) reduced platelet presence when compared to untreated injured heart (**Figures 6C, D**).

Multiphoton imaging detected neutrophil presence even within the deeper layers of the injured left ventricle, although their presence was highest in the outermost layer and decreased with depth. A significant (p<0.05) increase in neutrophil presence with heparin alone was again noted when compared to untreated injured hearts, as was observed intravitally. Whilst cellular therapy alone significantly (p<0.01) decreased their presence, dual therapy significantly (p<0.05%) decreased this further when compared with cellular therapy alone (**Figures 6E, G**). Adherent platelets were also present throughout the myocardium with the greatest presence in the outermost layer. A significant (p<0.01) decrease was noted in mice receiving heparin alone but no difference was observed in mice receiving only cellular therapy. However, platelet responses were most effectively (p<0.0001) reduced in mice receiving dual therapy (**Figures 6F, G**).

### Dual therapy most effective at reducing infarct size

Infarct size was large in injured hearts at 49.59 ± 5.85% (as a % of the AAR). Heparin alone slightly reduced infarct size to 41.48 ± 4.6%. 3D cultured MSCs-Y201 significantly (p<0.01) reduced infarct size to 27.25 ± 2.14% when compared to mice receiving no treatment. However, dual therapy significantly (p<0.001) reduced infarct size further to 23.69 ± 3.95% (**Figures 6H, I**).

### 3D cultured MSCs-Y201 most effective at reducing endothelial oxidative damage, adhesion molecule expression and circulating serum levels of multiple pro-inflammatory factors

**Figure 7:**
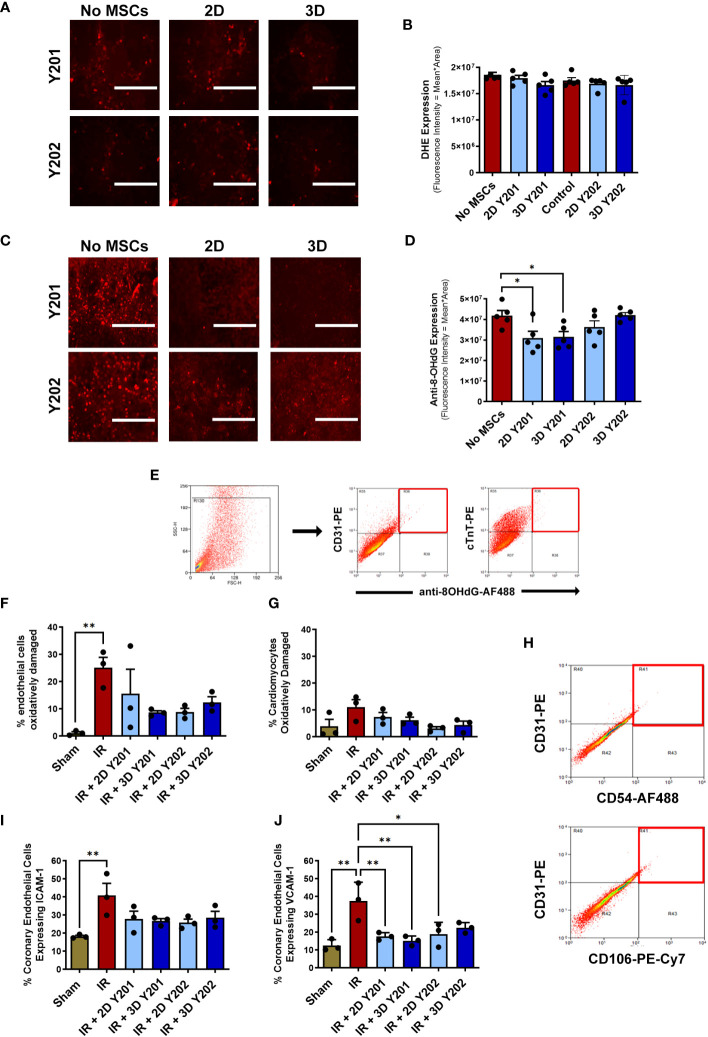
Effects of 2D/3D culture of MSCs on endothelial and cardiomyocyte oxidative damage and adhesion molecule expression. **(A)** Representative images and **(B)** quantitative flow cytometric analysis of the DHE staining (red) in H_2_O_2_ treated VCECs co-cultured +/- MSCs for 24 hours. **(C)** Representative images and **(D)** quantitative flow cytometric analysis of the oxidative injury marker 8-OHdG (red) in H2O2 treated VCECs co-cultured +/- MSCs for 24 hours. **(E)** Representative scatter plots demonstrating the selective gating strategy used to identify CD31^+^ coronary ECs and cTnT+ cardiomyocytes in digested heart samples. Oxidative damage detected using an anti-8-OHdG antibody (red squares) at 2 hours reperfusion. Quantitative flow cytometric analysis of oxidative damage in **(F)** coronary ECs and **(G)** cardiomyocytes. **(H)** Representative scatter plots demonstrating the selective gating strategy used to identify CD31^+^ coronary ECs in digested heart cells. Adhesion molecules detected using anti-ICAM-1 / anti-VCAM-1 antibodies (red squares). Quantitative flow cytometric analysis of **(I)** ICAM-1 and **(J)** VCAM-1 expression on coronary ECs. Results presented as mean ± SEM. *p<0.05, **p<0.01 with 2-way ANOVA and Sidak’s multiple comparison test. N=3-5 per group with 25,000 events analysed flow cytometrically. Scale bar = 200um.

Neither 2D or 3D cultured MSCs-Y201 or MSCs-Y202 modified ROS generation from VCECs when compared to VCECs cultured in the absence of MSCs (**Figures 7A, B**). Despite this, 2D and 3D cultured MSCs-Y201 significantly (p<0.05) decreased the fluorescence intensity of 8-OHdG staining in VCECs when compared to VCECs cultured in the absence of MSCs (**Figures 7C, D**). Flow cytometric analysis of digested heart coronary ECs demonstrated that oxidative injury was significantly (p<0.01) higher in injured hearts by 2 hours post-reperfusion compared to sham hearts. Indeed, 25.07 +/- 3.88% of the total ECs analysed underwent oxidative damage after injury compared to 1.12 +/- 0.48% in sham hearts. Interestingly, all MSC sub-populations, regardless of culture technique, reduced EC oxidative damage following injury, with the greatest reduction noted with 3D cultured MSCs-Y201 (**Figures 7E–G**). Only 11.10 +/- 2.77% of cardiomyocytes underwent oxidative damage following IR injury compared to 3.89 +/- 2.63% of sham heart cardiomyocytes. This was not statistically significant nor changed by any MSC therapy (**Figures 7F, G**). Flow cytometric analysis of ICAM-1 and VCAM-1 on digested heart coronary ECs demonstrated significantly (p<0.01) increased expression in injured hearts when compared to sham hearts. All MSC sub-populations, regardless of culture technique, reduced ICAM-1 and VCAM-1 expression, with the greatest reduction in VCAM-1 observed with 3D cultured MSCs-Y201 (**Figures 7H–J**).

Nine pro-inflammatory cytokines were significantly (p<0.05) elevated in the serum of injured mice when compared to sham mice. 2D cultured MSCs-Y201 did not significantly reduce any of the 23 cytokines investigated. However, all other MSC groups were able to significantly (p<0.05-0.01) reduce between 3-9 pro-inflammatory cytokines when compared to untreated injured mice, with the greatest reduction noted in mice receiving 3D cultured MSCs-Y201 (**Table 1**).

**Table 1:** Effects of 2D/3D culture of MSCs on circulating serum levels of inflammatory cytokines.

Cytokines	Sham	IR injury	IR + 2D MSCs-Y201	IR + 3D MSCs-Y201	IR + 2D MSCs-Y202	IR + 3D MSCs-Y202
**Interleukin-1α**	14.07 ± 2.90	18.62 ± 3.95	13.22 ± 1.99	7.13 ± 1.87*	8.33 ± 0.79**	10.63 ± 2.04*
**Interleukin-1β**	37.18 ± 5.06	42.84 ± 4.37	49.79 ± 10.80	33.92 ± 3.40	40.70 ± 2.67	45.73 ± 7.10
**Interleukin-2**	14.92 ± 3.00	11.65 ± 0.86	17.10 ± 2.76	14.07 ± 1.04*	15.69 ± 0.88**	13.88 ± 1.96
**Interleukin-3**	17.13 ± 2.45	16.46 ± 1.67	13.68 ± 2.67	20.23 ± 1.91	10.72 ± 1.14**	14.84 ± 2.81
**Interleukin-4**	15.71 ± 3.34	13.46 ± 2.69	12.02 ± 3.02	17.43 ± 3.38	9.37 ± 2.59	19.54 ± 3.55
**Interleukin-5**	32.63 ± 11.95	31.56 ± 5.69	58.28 ± 19.28	19.42 ± 1.48*	62.24 ± 12.95*	78.14 ± 14.77**
**Interleukin-6**	351.11 ± 126.42	1993.53 ± 841.45*	8258.03 ± 3053.95	495.75 ± 95.47*	2169.67 ± 356.71	5555.47 ± 2012.10
**Interleukin-9**	51.29 ± 7.54	45.76 ± 4.83	39.92 ± 5.87	52.82 ± 4.97	33.22 ± 3.52*	28.76 ± 6.26
**Interleukin-10**	143.57 ± 9.06	222.70 ± 45.84*	202.09 ± 16.63	132.55 ± 11.73*	126.73 ± 5.33*	154.59 ± 10.41
**Interleukin-12(p40)**	2253.75 ± 350.50	14588.86 ± 5889.30*	2943.45 ± 246.52	1040.67 ± 54.70*	1930.09 ± 203.49*	2042.37 ± 222.78*
**Interleukin-12(p70)**	501.84 ± 85.43	555.36 ± 57.31	665.98 ± 90.92	845.47 ± 88.45**	487.45 ± 36.06	735.89 ± 122.67
**Interleukin-13**	278.182 ± 51.10	337.87 ± 38.67	338.73 ± 76.32	250.53 ± 25.51*	279.56 ± 28.81	326.23 ± 49.17
**Interleukin-17**	18.70 ± 2.68	14.50 ± 1.22	71.15 ± 21.59*	223.36 ± 24.34**	43.72 ± 11.59*	117.62 ± 45.04*
**CCL11 (Eotaxin)**	992.67 ± 113.83	1607.58 ± 50.43*	3690.49 ± 345.05**	1811.43 ± 125.62	2354.80 ± 94.37**	2944.77 ± 196.19**
**G-CSF**	395.23 ± 97.79	2720.72 ± 1272.80*	1345.84 ± 287.82	259.23 ± 42.33*	544.76 ± 79.95*	577.34 ± 57.34
**GM-CSF**	54.52 ± 7.25	61.95 ± 6.50	65.34 ± 11.55	55.94 ± 5.24	49.87 ± 3.42	61.36 ± 8.84
**IFNγ**	46.44 ± 7.28	42.25 ± 4.50	44.16 ± 5.34	67.00 ± 6.33**	33.50 ± 3.63	46.14 ± 9.68
**Cxcl1/KC**	326.15 ± 93.22	1769.48 ± 783.47*	1307.16 ± 285.36	289.87 ± 51.56*	901.31 ± 147.50	1180.94 ± 163.29
**CCL2/MCP-1**	638.92 ± 121.67	5016.62 ± 2117.35*	3795.82 ± 1505.67	514.98 ± 63.93*	1481.29 ± 62.80	2191.60 ± 513.48
**CCL3/MIP-1α**	21.84 ± 8.95	48.51 ± 21.69	25.12 ± 9.19	7.04 ± 1.09*	35.12 ± 11.01	21.80 ± 5.28
**CCL4/MIP-1β**	169.46 ± 56.60	1545.83 ± 736.81*	321.19 ± 132.70	72.36 ± 9.10*	138.52 ± 20.65*	178.29 ± 47.41*
**CCL5/RANTES**	108.06 ± 15.03	248.96 ± 79.09*	274.16 ± 16.38	200.72 ± 9.37	233.20 ± 25.34	257.36 ± 9.64
**TNFα**	191.95 ± 29.16	369.49 ± 95.21*	219.90 ± 32.44	349.34 ± 34.21	168.99 ± 16.04*	245.22 ± 46.77
**Total Increased**		10	2	4	4	3
**Total Decreased**		0	0	11	7	3

Blue: cytokines whose concentration significantly increased after myocardial IR injury when compared to sham mice. Green: cytokines whose concentration significantly decreased in MSC-treated groups compared to untreated injured mice. Yellow: cytokines whose concentration increased in MSC-treated groups compared to untreated IR injured mice. Boxed in red: well-known anti-inflammatory cytokines. Results presented as mean ± SEM pg/ml. *p<0.05 when compared to sham hearts; **p<0.01 when compared to sham hearts. N=3 per group.

## Discussion

Reperfusion injury post-PCI remains one of the top 10 unmet clinical needs in cardiology (29). Since the smallest coronary blood vessels are increasingly recognised as a primary target of reperfusion injury, therapeutic interventions must be able to protect these delicate structures and maintain adequate perfusion in the heart. We have previously demonstrated, using novel intravital imaging of the anaesthetised mouse beating heart, a number of microcirculatory perturbations post-reperfusion injury including increased neutrophil recruitment, increased microthrombus formation and decreased FCD (14). The current study tested whether cellular therapy using MSCs, known for their potent anti-inflammatory properties, could successfully maintain the viability of the coronary microcirculation in the beating heart *in vivo*. However, despite this inherent property, clinical success using MSCs for treating various conditions has been limited. Therefore, we investigated intravitally whether we could enhance their potential vasculoprotective efficacy through 3D culture and the testing of different BM-derived MSC sub-populations for their coronary homing and anti-neutrophil/anti-platelet capacity.

MSCs are routinely grown in easy and relatively inexpensive 2D cultures in which they adhere to plastic surfaces, spread, become flat and greatly increase their size. However, infusion of such large cells reduces their ability to circulate beyond a single pass and reach target sites of injury including the heart. Indeed, <5% of infused MSCs reach the injured heart with most becoming entrapped within pulmonary capillaries (30). The current study firstly demonstrated that MSCs cultured using a simple scaffold-free, hanging drop method clumped together and formed compact spheroids that were 50-60% smaller in diameter than MSCs grown using conventional 2D culture. This is consistent with studies in which cellular diameter decreases of up to 70% were observed with 3D culture (31, 32). It was anticipated that after infusion, these smaller 3D cultured MSCs would escape non-specific entrapment and maintain a circulating pool of MSCs, thereby allowing more efficient delivery to the heart (33). However, our novel intravital imaging of the beating heart demonstrated that whilst 3D culture increased numbers of MSCs-Y201 and MSCs-Y202 circulating through the coronary microcirculation, this was were only observed immediately after their infusion and not thereafter. This is consistent with our previous intravital findings in other organs where circulating haematopoietic stem/progenitor cells (HSPCs) were also only observed on ‘first pass’ (34–36). The initial increase immediately after infusion may be due a transient reactive hyperaemic response in the ischaemic ventricle upon reperfusion which may have delivered more MSCs to the heart (14). Additionally, increased circulation may be linked to the recovery of critical stem cell homing receptors, such as CXCR4 which are lost during conventional culture, with 3D culture. This was indeed the case for MSCs-Y201 and has been described by others (37–39). The recruitment of 3D cultured MSCs-Y201 in the heart was very limited and therefore not sufficient to significantly reduce pulmonary presence when compared to the other groups. Indeed, the vast majority of the half a million MSCs that were injected became non-specifically entrapped in the lungs with no cells detected in other non-cardiac organs (data not presented).

MSC aggregation into tightly packed clusters creates an *in vivo*-like microenvironment which better preserves their phenotype and innate properties (40). Whether this impacted their ability to adhere to endothelial counterligands was tested *in vitro* and in the beating heart. 3D culture consistently enhanced adhesion of only MSCs-Y201 in static adhesion assays but improved retention of both sub-populations *in vivo*, albeit in small numbers. Interestingly, 1,731 genes have been identified to be overexpressed in hanging drop cultured human MSCs when compared to monolayer cultured cells, including those encoding critical MSC surface integrins and receptors involved in adhesion (41). Furthermore, similar to our own data, others have also shown the ability of 3D spheroid culture to enhance expression of adhesion integrins on various cell types, including a 3-fold increased expression of the ICAM-1 ligand lymphocyte function associated antigen-1 (LFA-1) on cancer cells and the VCAM-1 ligand very late antigen-4 (VLA-4) on HSPCs (31, 42, 43). In the current study more uniform distribution of adhesion molecules was also noted on 3D cultured MSCs which, when noted on leukocytes, has been known to increase the effectiveness of their recruitment within injured sites (44). Collectively, these changes may mechanistically explain the observed increased adhesion of 3D cultured MSCs.

Despite 3D culture benefitting the adhesion of both MSC sub-populations equally *in vivo*, only 3D cultured MSCs-Y201 modified coronary microcirculatory perturbations. To date, MSCs derived from a single site such as the BM have not been enriched for a particular intra-tissue sub-population prior to their clinical use for CVDs. However, our novel results suggest that a significant barrier to realize the therapeutic potential of MSCs is their intrinsic heterogeneity despite their same origin and not necessarily their local coronary retention, which even after 3D culture was limited. An anti-neutrophil effect was significant 45 minutes post-infusion, but only with 3D cultured MSCs-Y201, decreasing neutrophil presence by approximately 30%. Whether there is a difference in the secretory profile of the two tested MSC sub-populations warrants further detailed investigation. However, it is well established that MSCs can secrete multiple anti-inflammatory factors which can suppress an overactivated immune system within cytokine-rich environments and thereby attenuate neutrophil infiltration. These include interleukin-10, transforming growth factor-β, inducible nitric oxide synthase, prostaglandin-E2 (PGE2) and indoleamine pyrrole 2,3-dioxygenase. MSCs can also suppress ROS secreted by neutrophils which would protect the endothelium with which neutrophils make close adhesive contact (45).

MSCs, as well as being anti-inflammatory, can also exhibit pro-inflammatory properties in certain instances such as to stimulate pathogen clearance (45). Indeed the MSCs used in the current study have been shown to have varying immunomodulatory ability based on the expression of CD317 (24, 25). MSCs-Y202 have been identified as nullipotent CD317^pos^ cells that make up approximately 30% (CD317^dim^ ∼28%; CD317^bright^ ∼2%) of the BM MSC population. These specific MSCs are less effective at suppressing T cell proliferation and more capable of polarising them towards a pro-inflammatory type 1 T-helper (Th1) subset than MSCs-Y201. In contrast, CD317^neg^ MSCs-Y201, making up 70% of the BM MSC population, polarise T cells towards an anti-inflammatory type 2 T-helper (Th2) subset (25). CD317, also known as tetherin is a transmembrane protein best known for its ability to inhibit the spread of viral infection by ‘tethering’ them to the cell membrane. In addition, tetherin activates nuclear factor kappa B (NF-*κ*B), a transcriptional activator that leads to the rapid expression of pro-inflammatory cytokines, thus contributing to antiviral defence (46). We therefore speculate that when a mixed population of BM-derived MSCs are infused for therapeutic purposes, the pro-inflammatory CD317^pos^ sub-population may ‘dilute’ the anti-inflammatory benefits of the CD317^neg^ sub-population and may even be harmful (25). Although this particular marker may molecularly identify a MSC subset with specific vasculoprotective properties in the injured heart, we show that their expansion in 3D cultures also remains equally important.

We have previously demonstrated that capillary occlusion by platelet aggregates is a key contributor to decreased perfusion in the IR injured heart (14). Since 3D cultured MSCs-Y201 did not significantly modify platelet behaviour, we investigated whether co-transplantation with an anti-coagulant could modify both platelet and neutrophil behaviour as previously demonstrated in the colon of colitis mice (47). Heparin was chosen as it is a widely used, safe drug which could be used clinically as an adjuvant if it was noted to improve MSC therapy. We firstly showed that heparin alone led to a gradual but worrying increase in neutrophil recruitment, an effect observed by others in different injuries such as in the peritonitis model (48, 49). It is plausible that heparin did indeed prevent microthrombus formation, unblocked occluded capillaries and thus improved perfusion. This may have consequently increased neutrophil trafficking and therefore their adhesion within a primed coronary microcirculation. However, dual therapy with heparin, whilst not improving the local presence of 3D cultured MSCs-Y201 (data not shown), did accelerate their anti-neutrophil ability, further decreased platelet presence and reduced infarct size more so than single therapy with MSCs alone. In this scenario, we postulate that heparin would have indirectly reduced microthrombus formation and the MSCs would have prevented any IR- and heparin-mediated neutrophil adhesion. Heparin can indirectly modify microthrombi presence through its actions as an anti-coagulant that reduces thrombin activity and consequently fibrin deposition. Lack of fibrin reduces the structural stability, strength and adhesive surfaces provided to platelet aggregates. Furthermore, the lack of thrombin, which is a highly potent platelet agonist, would also lead to reduced platelet activation and aggregation.

It is worth highlighting that the use of a single anti-coagulant therapy versus single cellular therapy versus dual therapy resulted in a 16%, 44% and 52% reduction in infarct size respectively. Although dual therapy did not significantly reduce infarct size statistically compared to cellular therapy alone, the modest salvage of an extra 8% more viable myocardium would have biological significance. Indeed, a 5% increase in infarct size observed clinically is independently associated with a 19% increase in mortality at 1-year (50). Furthermore, Pride and colleagues found clinically that every 5% increase in infarct size was associated with a 6.1% decrease in ventricular ejection fraction which correlated with poorer prognosis (51). Hence, we believe that even seemingly small improvements in infarct size can profoundly impact outcomes even if they fail to reach statistical significance (51, 52). Hence, overall our novel data suggested that 3D culture of the MSC-Y201 sub-population was superior in its ability to reduce inflammatory and thrombotic cell presence, improve flow and decrease infarct size after myocardial IR injury, with heparin co-administration providing further benefit at the level of the coronary microvasculature and infarct size.

Potential mechanisms by which MSCs-Y201 conferred vasculoprotection were also investigated. Oxidative stress following MI is associated with injury to the microvasculature which, through damage and denudation of ECs, can rapidly increase neutrophil and platelet activity. Indeed, we noted a remarkable increase in oxidative injury in 25% of coronary ECs when assessed flow cytometrically. Moreover, the coronary endothelium, rather than cardiomyocytes, appeared to be the primary and initial target of oxidative injury confirming our previous findings (14). Others have also shown this earlier and increased susceptibility of ECs to IR injury when compared to cardiomyocytes, with apoptotic death occurring at 5 and 60 minutes for ECs and cardiomyocytes respectively in isolated perfused hearts (53, 54). Collectively, these studies highlight the coronary microcirculation as an early and principal therapeutic target to prevent ensuing muscle damage. Growing evidence suggests MSCs can indirectly exert anti-oxidant effects through release of superoxide dismutase (SOD), catalase and glutathione peroxidase. For example, MSCs have recently been shown to enhance, rather than release themselves, SOD from cells exposed to oxidative stimuli *in vitro* and also in murine models of inflamed gut and ageing (55). Preventing EC damage may potentially explain their ability to reduce the recruitment of thromboinflammatory cells. It was therefore exciting to see that all MSC sub-populations could rapidly reduce the burden of oxidative stress on coronary ECs *in vivo* with MSCs-Y201 being most effective *in vitro* where they maintained an anti-oxidant effect even after 24 hours of H_2_O_2_ exposure.

The initial telomere length of the donor’s MSCs could therefore be a limiting factor of the MSCs therapeutic potential since it will affect the survival and integration ability of the trans-planted cells to the adult tissue The initial telomere length of the donor’s MSCs could therefore be a limiting factor of the MSCs therapeutic potential since it will affect the survival and integration ability of the trans-planted cells to the adult tissue.

It is well established that neutrophil adhesion to activated ECs is facilitated by endothelial expression of ICAM-1 and VCAM-1 with expression of the latter noted in res[ponse to oxidative damage (56). We confirmed previous findings that myocardial IR injury significantly increased both on CD31^+^ ECs in IR injured hearts. Interestingly, all MSC groups tested were able to reduce the overexpression of both ICAM-1 and VCAM-1, with greater and significant responses observed for the latter. Again, it seems that an ability to rapidly modify inflammatory markers *in vivo* appears to be an innate capability of BM-derived MSC subpopulations and is supported by other studies. Although there was no significant difference in the *in vivo* anti-oxidant and anti-adhesion molecule ability of all 4 MSC groups, 3D cultured MSCs-Y201 consistently demonstrated itself to be the most effective. These multiple beneficial effects at a molecular level may explain why this particular MSC group was able to significantly tame neutrophil recruitment within the injured beating heart *in vivo*. Similar reductions in endothelial ICAM-1/VCAM-1 have been observed by different groups, albeit using immunostaining and rt-PCR methods. Qui and colleagues showed a reduction of endothelial ICAM-1 expression after human umbilical cord MSC treatment in a renal model of IR injury. Rahbarghazi and colleagues more recently stated that there was limited data on the modulatory effects of MSCs on the expression levels of ICAM-1 and VCAM-1 in lung tissues and so investigated this in rat asthmatic models. They also found BM-MSCS decreased expression of both on pulmonary endothelium (57). In their study, they further tested whether this benefit could be conferred via a paracrine effect by performing parallel experiments using BM-MSC conditioned media. Although conditioned media was effective, the ability to decrease adhesion molecule expression was more evident in the cell-treated group.

2D cultured MSCs-Y201 did not significantly decrease any circulating serum cytokine levels in IR injured mice. It was therefore remarkable that simply culturing these specific cells in hanging drops dramatically converted them into the most effective cellular therapy at reducing pro-inflammatory cytokine levels. Further studies are required to determine how these decreases were elicited. However, preconditioning MSCs through 3D culture has previously been shown to modify their secretome such that there is an increased release of anti-inflammatory factors including PGE2 and TNFα stimulated gene-6 (TSG-6) which could subsequently suppress inflammatory cytokine secretion through paracrine actions on various cells (31, 58). An interesting observation in the current study was the universal ability of all MSC sub-populations to decrease to some degree levels of IL-1α, IL-12(p40) and CCL4/MIP-1β. It is possible therefore that targeting these specific inflammatory cytokines is a critical vasculoprotective mechanism. Although Shin and colleagues recently demonstrated that the secretome profile of MSCs from different sites varied, further investigation is required to determine if this is also the case for MSC sub-populations derived from the same site (59). Whilst endothelial oxidative damage and inflammatory adhesion molecule expression was equally impacted by 2D and 3D-cultured Y201 MSCs, the most striking observation was this differential effect on circulating inflammatory cytokines between 2D and 3D cultured MSCs-Y201. This effect, alongside the ability of 3D cultured MSCs-Y201 to preferentially reduce local neutrophil recruitment and improve FCD, appears to be the most likely explanation underlying their observed cardioprotection rather than direct beneficial effects on the coronary ECs *per se.* However, myocardial IR injury is a multi-faceted injury and therefore likely benefits from 3D cultured MSCs-Y201 exerting multiple protective mechanisms simultaneously to confer cardioprotection including effects on the microvessels themselves.

Only a limited number of studies have compared the benefits of 2D and 3D cultured MSCs in preclinical studies and currently no trials have yet evaluated their clinical efficacy. One of the major limitations of taking this approach to the clinic is the laborious preparation required to generate 3D spheroids of MSCs. This significantly limits their large-scale production for translational purposes. To circumvent this issue, future studies could consider testing the coronary vasculoprotective efficacy of MSCs-Y201 cultured on ultra low-attachment surfaces. These plates are coated in a way that inhibits immobilisation, forcing cells into a suspended state and thereby enabling spheroid formation. Importantly, they offer a less laborious technique for culturing MSCs as spheroids. Alternatively, increasing our knowledge of the paracrine factors released in the secretome from 3D cultured MSCs-Y201, and specifically identifying and testing proteins with vasculoprotective potential, would also be a worthwhile pursuit. Our study demonstrates that co-administration of heparin improved the vasculoprotective capacity of 3D cultured MSCs-Y201. Hence, future studies could also assess whether dual therapy of MSCs with other pharmacological drugs, such as contemporary anti-platelet drugs already used to treat patients post-PCI, may further improve their beneficial effects at the level of the coronary microcirculation.

To conclude, further improvements to MI patient outcomes will depend on addressing problems in the coronary microcirculation that arise during reperfusion. Therefore, developing strategies specifically directed at restoring microvascular function is of paramount importance. MSC cytotherapy is a promising strategy but clinical success has been limited. We show that 3D culture of BM-derived MSCs, a simple non-invasive and non-genetically engineered approach, improves their vasculoprotective ability without remarkably increasing their local presence in the IR injured heart. Moreover, 3D culture of a specific CD317^neg^ MSC sub-population, in combination with heparin, could offer a powerful new cellular treatment for improving multiple perturbations in the coronary microvessels post-reperfusion.

## Data availability statement

The raw data supporting the conclusions of this article will be made available by the authors, without undue reservation.

## Ethics statement

The studies involving humans were approved by REC 07/Q1105/9; University of York. The studies were conducted in accordance with the local legislation and institutional requirements. The participants provided their written informed consent to participate in this study. The animal study was approved by UK Home Office and University of Birmingham Animal Welfare and Ethical Review Body (AWERB). The study was conducted in accordance with the local legislation and institutional requirements.

## Author contributions

KB: Formal Analysis, Writing – original draft, Data curation, Methodology. DK: Writing – review & editing. PG: Writing – review & editing, Resources. NK: Writing – review & editing, Conceptualization, Formal Analysis, Funding acquisition, Project administration, Writing – original draft.
